# Tree species richness decreases while species evenness increases with disturbance frequency in a natural boreal forest landscape

**DOI:** 10.1002/ece3.1944

**Published:** 2016-01-18

**Authors:** Daniel Yeboah, Han Y.H. Chen, Steve Kingston

**Affiliations:** ^1^ Faculty of Natural Resources ManagementLakehead University955 Oliver RoadThunder BayONP7B 5E1Canada; ^2^ Ontario Ministry of Natural Resources435 James Street South, Suite 221DThunder BayONP7E 6S7Canada

**Keywords:** Climate, intermediate disturbance hypothesis, soil drainage class, species diversity, structural equation models, time since fire

## Abstract

Understanding species diversity and disturbance relationships is important for biodiversity conservation in disturbance‐driven boreal forests. Species richness and evenness may respond differently with stand development following fire. Furthermore, few studies have simultaneously accounted for the influences of climate and local site conditions on species diversity. Using forest inventory data, we examined the relationships between species richness, Shannon's index, evenness, and time since last stand‐replacing fire (TSF) in a large landscape of disturbance‐driven boreal forest. TSF has negative effect on species richness and Shannon's index, and a positive effect on species evenness. Path analysis revealed that the environmental variables affect richness and Shannon's index only through their effects on TSF while affecting evenness directly as well as through their effects on TSF. *Synthesis and applications*. Our results demonstrate that species richness and Shannon's index decrease while species evenness increases with TSF in a boreal forest landscape. Furthermore, we show that disturbance frequency, local site conditions, and climate simultaneously influence tree species diversity through complex direct and indirect effects in the studied boreal forest.

## Introduction

The relationships between disturbance and plant species diversity have been studied for decades, since Connell (1978) proposed the intermediate disturbance hypothesis (IDH), which predicts plant species diversity to peak at intermediate levels of disturbance frequencies, intensities, and extents (i.e., area disturbed). However, the patterns of diversity and disturbance relationships and the possible mechanisms driving those patterns remain the subject of debate (Mackey and Currie 2001; Shea et al. 2004; Svensson et al. 2012). For example, Mackey and Currie (2001) reviewed 116 species richness‐, 53 Shannon's index‐, and 28 evenness‐disturbance relationships in studies published from 1985 through 1996 and found support for the IDH in only 16% of richness, 19% of Shannon's index, and 11% of evenness relationships, respectively. Theoretical research has even concluded that the IDH should be abandoned because the three proposed mechanisms that support IDH are logically invalid (Fox 2013). However, recent field studies continue to provide evidence of support for the IDH in tropical (Bongers et al. 2009) and boreal forests (Mayor et al. 2012). Patterns of diversity and disturbance relationships are more frequently reported to be positively or negatively linear than the IDH predicted hump‐shaped relationship (Mackey and Currie 2001). The negative linear relationship is consistent with initial floristic composition hypothesis, which states that all species occur immediately after disturbance and temporal changes in diversity and succession are driven by local extinction of species from overstory vegetation through differential rates of growth, competition, and longevity (Egler 1954).

There are several reasons for the different outcomes in the testing of the IDH. Firstly, the range of disturbance frequency and intensity may influence the outcomes. For example, if only the range from high to intermediate disturbance frequencies are considered in the IDH, a positive diversity and disturbance relationship would be expected. The positive relationship, however, would be inappropriate in rejecting the IDH (Mackey and Currie 2001). Secondly, the relationships between different measures of diversity and disturbance may differ (Mackey and Currie 2001; Zhang et al. 2014). Despite the crucial importance of understanding the role of species evenness in ecosystem function (Hillebrand et al. 2008; Zhang et al. 2012), substantially fewer studies have examined species evenness‐disturbance relationships in natural systems, and mechanisms responsible for species presence and absence and their relative abundance may differ with forest stand development (Chen et al. 2009; Chen and Taylor 2012). Lastly, diversity is strongly influenced by climatic factors (Francis and Currie 2003) and local site conditions (Huston 1993; Zhang et al. 2014). Failing to consider the influences of climatic factors and local site conditions may also contribute to the outcomes of testing the IDH.

Some of the disparities in disturbance–diversity relationships (DDR) may be resolved by considering the multiple interacting mechanisms that influence plant coexistence in natural forests (Shea et al. 2004; Grace et al. 2007). Stand‐replacing fire is widespread, particularly in the boreal forest (Bowman et al. 2009). Time since fire (TSF) plays an important role in influencing plant coexistence, succession, and/or other ecosystem processes (Johnson 1996; Grandpré et al. 2000; Clark et al. 2003; Wardle et al. 2003). Fire regimes in boreal forest vary spatially due to local site conditions, such as soil drainage, which imposes differential fuel moisture levels among locations, and thus moderate the spread of fire (Larsen 1997; Cyr et al. 2007; Mansuy et al. 2010). Fire regimes are also influenced by climate such as changes in mean annual temperature and precipitation (Parisien et al. 2011). Given the importance of multiple ecological interactions (Grace et al. 2007), testing the DDR requires untangling the multiple mechanisms that influence plant diversity.

Here, we examined the relationships between species richness, Shannon's index, evenness, and TSF in the central boreal forest, where stand‐replacing fire is frequent (Senici et al. 2010, 2013). We examined how each measure of diversity responded to TSF by simultaneously accounting for the effects of climate and local site conditions. We also explored the multiple relationships among tree species diversity, TSF, local site conditions, and mean annual temperature using structural equation models (SEMs). In the SEMs, we tested (1) the effects of temperature and local site conditions on TSF and (2) the effects of TSF, local site conditions, and temperature on species richness, Shannon's index, and evenness. Since most tree species can re‐establish immediately following fire in the western and central boreal forest of North America (Gutsell and Johnson 2002; Chen et al. 2009; Ilisson and Chen 2009), we hypothesize species richness to decrease with TSF since local extinction may occur for early‐successional, shade‐intolerant species such as *Pinus banksiana* that are incapable of regenerating under canopy (Chen and Popadiouk 2002). We predict that Shannon's index will decrease with TSF, suggested by the theory of initial floristic composition (Egler 1954). We also hypothesize species evenness to increase with TSF because dominance of early‐successional species tends to decrease with stand development (Bergeron 2000; Chen and Taylor 2012; Bergeron et al. 2014).

## Materials and Methods

### Study area

**Figure 1:**
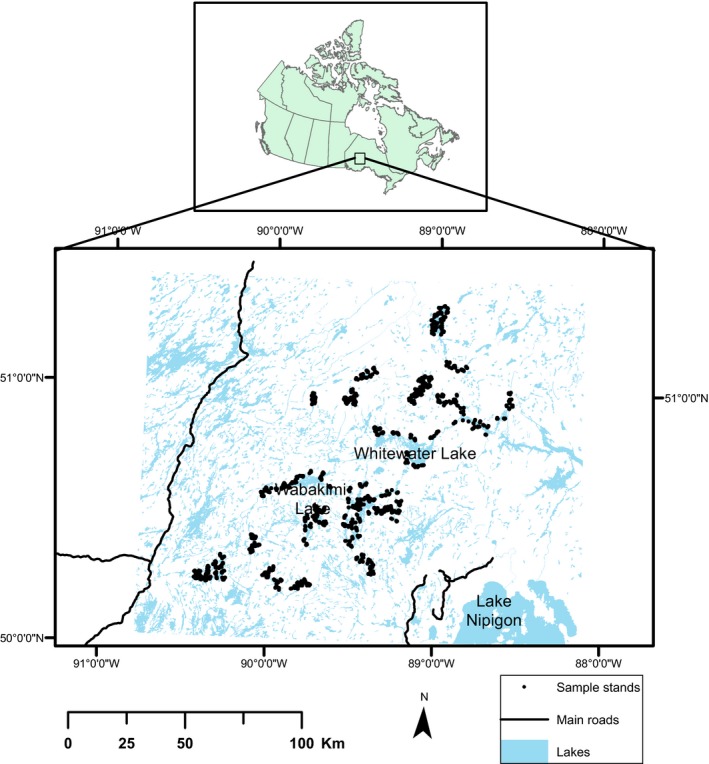
The study plots (*n* = 1018) located in northwestern Ontario, Canada.

This study was located in Wabakimi Provincial Park in northwestern Ontario (Fig. 1). The study area is a remote wilderness park with minimal road access, virtually no human activity, and the absence of commercial forest harvesting. Hence, the park provides an ideal landscape to test the effects of relatively natural fire frequency on species diversity. This park is the second largest in Ontario, covering a total area of 892,000 ha and situated within the boundaries of 50°00′N to 51°30′N and 90°30′W to 88°30′W. Mean annual temperature and annual precipitation recorded, from 1971 to 2010, at the nearest climate station in Armstrong, were −1.3°C and 700 mm, as suggested by Environment Canada (2011). Elevation is between 328 and 462 m above sea level (Soil Landscapes of Canada Working Group, 2010). Soils consist of sand, silt, and clay types, and the predominate soil orders are Brunisol and Podzol (Soil Landscapes of Canada Working Group, 2010). Common tree species within the park include the following: *Pinus banksiana* Lamb., *Populus tremuloides* Michx., *Populus balsamifera* (L.), *Betula papyrifera* March., *Picea mariana* (Mill) Britton, *Picea glauca* (Moench) Voss, *Abies balsamea* (L.) Mill., and tamarack [*Larix laricina* (Du Roi) K. Koch].

### Sampling strategy

Stratified random sampling was employed to quantify the forest composition and productivity associated with diverse local site conditions and stand age classes based on interpreting aerial photographs, by the Ontario Ministry of Natural Resources. A total of 1018 sample plots were spatially interspersed across the park with a distance of at least 500 m between the closest sampled plots, in order to avoid the effects of spatial autocorrelation (Legendre and Legendre 2012). For each plot, a transect of 200 m was laid, and prism sweeps were taken at ten points of 20 m apart using a wedge prism with a basal area factor of two. At every sampling point, living trees with diameter at breast (dbh, 1.3 m above root collar) ≥10 cm were tallied by tree species and used to determine the stand basal area and tree species composition (Avery and Burkhart 2002).

### Species diversity

We considered diversity indices including tree species richness, Shannon's index, and species evenness. Species richness pertained to the number of species that were observed in each sample plot. Shannon's index was calculated by utilizing the basal area proportions of the constituent species within each sample plot. We used the inverse of Simpson's dominance index as the measure of species evenness, which is considered to be independent of species richness (Smith and Wilson 1996). The evenness index was also determined using the basal area proportions of the constituent species.

### Explanatory variables

To understand DDR, we assessed the effects of TSF, mean annual temperature, average annual precipitation, and local site conditions on species richness, Shannon's index, and evenness. Disturbance frequency was measured as TSF (age in years) as originally proposed by Connell (1978). The TSF was determined by using either fire data from Ontario fire history maps, which document all fires ≥200 ha since 1921, or by coring trees. We used these fire records to initially determine TSF for fires that occurred since 1921. Because of potential inaccuracy of fire maps due to escaped patches, as well as the lack of records for fires <200 ha, field validation was conducted by coring the dominant trees to the pith at the dbh from three dominant trees (Bergeron 1991; Senici et al. 2010). In cases where there was a discrepancy in the TSF between the fire map and the ring counting, we used TSF estimated from the latter approach for analyses. For fires occurring before 1921, we used tree ages to determine TSF. In the field, tree species known to regenerate immediately postfire (Bergeron and Brisson 1990) were preferentially selected in the following order: *Pinus banksiana*,* Populus tremuloides, Betula papyrifera*, and *Picea mariana* (Chen and Taylor 2012). All acquired cores were returned to the laboratory where the rings were counted under a dissecting microscope until an identical count was obtained in triplicate. From the tree ring counts, TSF was determined by adding 7, 8, or 17 years when ring counts were from *Pinus banksiana*,* Populus* sp. (or *Betula papyrifera*), or *Picea mariana* (Vasiliauskas and Chen 2002). For stands dominated by shade‐tolerant species such as *Picea glauca* or *Thuja occidentalis*, it was assumed that the trees of shade‐intolerant species present at the sites did not regenerate immediately postfire. In such cases, the age of the oldest tree was used regardless of the tree species as an approximation of the stand age (Senici et al. 2010). The mean stand age was 89 years (Table 1).

**Table 1:** Characteristics of study plots sampled (*n* = 1018) in the Wabakimi Provincial Park of Canada

Characteristic	Mean	Minimum	Maximum
Species richness	3.14	2	8
Shannon's index	0.66	0.05	1.80
Species evenness	0.61	0.20	0.99
TSF	89.00	20	209
MAT	−0.29	−0.83	0.42
MAP	711.40	701.0	724.8
SDC	1	0	9

TSF, time since fire (years); MAT, mean annual temperature (°C); MAP, mean annual precipitation (mm); SDC, soil drainage class (median is reported instead of mean).

Soil drainage class (SDC), which is comparable to soil moisture regime and nutrient regime classification (Chen et al. 2002), was used to represent local site conditions. SDC represents a composite measure of overall site quality, which is assessed from soil texture, soil thickness, and topographic position, soil permeability, depth of water table, and organic layer depth. SDC was determined on site using soil pits to the parent material, or 120 cm deep. SDC was ranked from 0 to 9, which represent dry, moderately fresh, fresh, very fresh, moderately moist, moist, very moist, moderately wet, wet, and very wet soil, respectively.

To determine the effects of temperature and precipitation on species diversity, we derived long‐term (1921–2010) climate estimates from BioSIM R: produced in Quebec, Canada (https://cfs.nrcan.gc.ca/projects/133), which generates scale‐free climate estimates based on latitude, longitude, and elevation (Hogg 1997). The climate estimates were used to calculate mean annual temperature (MAT) and mean annual precipitation (MAP).

### Statistical analysis

We developed individual models for measures of diversity using TSF, SDC, MAT, and MAP as predictors. We employed generalized linear model and boosted regression trees (BRT) to examine the effects of these predictors on diversity indices. Both modeling approaches yield similar results. For simplicity, we reported the results from the generalized linear models.

We also constructed separate SEM models for diversity indices. SEM possesses a unique strength for analyzing complex relationships, in that the same variable may be treated as a predictor and as a response variable (Grace et al. 2007, 2010). Goodness of fit for the model was determined from the maximum‐likelihood chi‐square test, and the model was judged as having a good fit if *P *> 0.05, which indicates that the model is consistent with the data (Rosseel 2012). The chi‐square test can be influenced by sample size; therefore, we also reported the comparative fit index (CFI) which is least affected by sample size (Bentler and Bonett 1980; Bentler 1990; Rosseel 2012). In a preliminary model, we included MAP as a predictor in SEMs; however, the model did not yield a good fit because *P *<* *0.05 and CFI < 0.9. Accordingly, as recommended (Grace et al. 2007, 2010), we modified SEMs to include the effects of MAT and SDC, on TSF, and TSF, MAT, and SDC on diversity, with or without the quadratic term for SDC. We treated SDC as a regular numeric variable in SEMs since SDC is an ordinal variable and we were interested whether species diversity could be quadratically related to SDC (Rosseel 2012; Zhang and Chen 2015). We determined the magnitude of direct effect from SEM coefficients. We also estimated the total effects of a given exogenous variable on different measures of diversity by adding standardized direct and indirect effects. The statistical significance for the SEM coefficients was evaluated using a bootstrap method, as bootstrapped estimates do not assume any particular distribution and thus are often suitable for non‐normal data such as the number of species (Bollen and Stine 1992).

## Results

**Figure 2:**
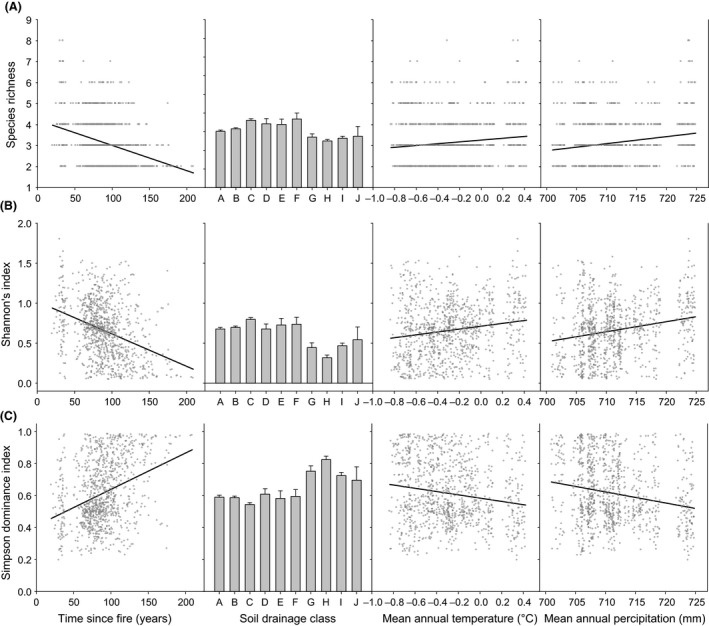
Bivariate relationships between diversity indices and time since fire, soil drainage class, mean annual temperature, and mean annual precipitation. (A) Species richness. (B) Shannon's index. (C) Species evenness. Soil drainage classes from A to J represent dry, moderately fresh, very fresh, moderately moist, moist, very moist, moderately wet, wet, and very wet soil, respectively. Values in the figures associated with soil drainage classes are mean + 1 SEM. Dots and lines in other figures are observed values and fitted linear regressions.

The final generalized linear models explained the 18%, 21%, and 21% variation of tree species richness, Shannon's index, and evenness, respectively (Table 2). TSF and SDC were the strongest predictors, whereas MAT and MAP were less important in all models (Table 2). Species richness decreased with TSF, increased with MAT and MAP, and was higher in intermediate SDC (Fig. 2). Species evenness, however, increased with TSF, decreased with MAT and MAP, and was higher in very moist and moderately wet sites than other SDCs. Shannon's index had similar relationships to the predictors as species richness (Fig. 2).

**Table 2:** Percent variance explained by time since fire (TSF, years), soil drainage class (SDC), mean annual temperature (°C), and mean annual precipitation (mm) on tree species richness, Shannon's index, and species evenness (*n* = 1018). Percent variance explained by each individual predictor is calculated as the sum of squares associated with the predictor divided by the total sum of squares for each model. The reported models including TSF, SDC, MAT, and MAP as predictors are better than the models with a quadratic term of TSF as an additional predictor based on Akaike information criterion; for all diversity indices, the quadratic term of TSF was statistically insignificant (*P *> 0.05)

Diversity index	TSF	SDC	MAT	MAP	Error distribution	*R* ^2^
Richness	11.13	5.53	0.01	0.86	Poisson	0.18
Shannon's index	11.72	8.52	0.07	0.78	Gaussian	0.21
Evenness	12.42	7.77	0.11	1.01	Gaussian	0.21

**Figure 3:**
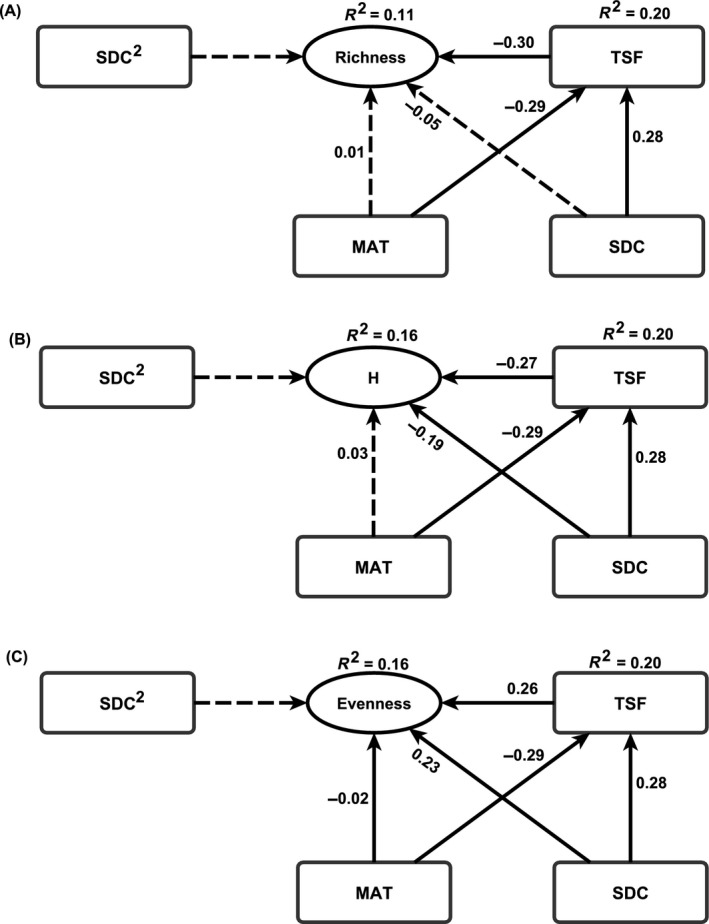
Results of structural equation modeling (SEM) relating tree species diversity to disturbance frequency. Solid lines represent significant (*P *< 0.05) SEM coefficients and dashed lines represent insignificant ones (*P* ≥ 0.05). (A) Species richness. (B) Shannon's index (H). (C) Species evenness. TSF, MAT, and SDC represent time since last fire (years), mean annual temperature (°C), and soil drainage class, respectively.

The SEM models with the quadratic term of SDC yielded an inadequate fit of data to the model for species richness (*P *< 0.001, df = 1, CFI = 0.931), Shannon's index (*P *< 0.001, df = 1, CFI = 0.938), and evenness (*P *< 0.001, df = 1, CFI = 0.938). As recommended (Grace et al. 2010), these models were modified by eliminating nonsignificant direct effects of the quadratic term of SDC. The modified model yielded adequate fit of data for species richness (*P *> 0.05, CFI = 1), Shannon's index (*P *> 0.05, CFI = 1), and evenness (*P *> 0.05, CFI = 1). There was a significantly negative direct effect of TSF on richness and Shannon's index, but a positive effect on evenness (Fig. 3). MAT had a negative direct effect and SDC had a positive direct effect on TSF in the richness model (Fig. 3). SDC had a negative indirect influence on species richness through TSF (Table 3).

**Table 3:** The direct, indirect, and total standardized effects on tree species richness, Shannon's index, and evenness based on structural equation models (SEM). The total effect was estimated by adding standardized direct and indirect effects

SEM model	Predictor	Pathway to each component of diversity	Effect	*P*‐value
Model for species richness in Fig. 3A	Time since fire	Direct	−0.3	<0.001
		Indirect effect	–	–
		Total effect	−0.3	<0.001
	Soil drainage class	Direct	−0.05	0.103
		Indirect effect through time since fire	−0.1	<0.001
		Total effect	−0.15	<0.001
	Mean annual temperature	Direct	0.01	0.887
		Indirect effect through time since fire	0.11	<0.001
		Total effect	0.12	<0.001
Model for Shannon's index in Fig. 3B	Time since fire	Direct	−0.27	<0.001
		Indirect effect	–	–
		Total effect	−0.27	
	Soil drainage class	Direct	−0.19	<0.001
		Indirect effect through time since fire	−0.1	<0.001
		Total effect	−0.29	<0.001
	Mean annual temperature	Direct	0.05	0.121
		Indirect effect through time since fire	0.12	<0.001
		Total effect	0.17	<0.001
Model for species evenness in Fig. 3C	Time since fire	Direct	0.26	<0.001
		Indirect effect	–	–
		Total effect	0.26	<0.001
	Soil drainage class	Direct	0.23	<0.001
		Indirect effect through time since fire	0.09	<0.001
		Total effect	0.32	<0.001
	Mean annual temperature	Direct	−0.05	0.114
		Indirect effect through time since fire	−0.11	<0.001
		Total effect	−0.16	<0.001

## Discussion

Despite a wide range of stand ages included in our study, our analysis demonstrated that species richness of overstory trees decreased with time since fire in the studied boreal forest. Based on the prediction of IDH, a recently disturbed forest stand would consist of early‐successional species, and shade‐tolerant species would grow into the stand, and eventually outlast early‐successional species. This succession process would result in young stands consisting of early‐successional species, old stands consisting of late‐successional species, and intermediate aged stands having both early‐ and late‐successional species diversity peaks in intermediate aged stands. However, in our boreal forests, most tree species can re‐establish immediately following fire (Gutsell and Johnson 2002; Chen et al. 2009). The decrease of species richness over time is attributable to age‐dependent local extinction of short‐lived early‐successional, shade‐intolerant species (Chen and Popadiouk 2002; Luo and Chen 2011).

By contrast, other empirical studies conducted in forests with long stand‐replacing disturbance intervals indicate that species richness and/or Shannon's index peaks at intermediate stand age (Zhu et al. 2009; Zhang et al. 2014). These contrasting findings appear to be attributable to long‐term ecosystem‐specific adaptive responses to disturbance frequencies: In fire‐frequent western and central boreal forests of North America (Weir et al. 2000; Senici et al. 2010), evolutionary selection has resulted in a pool of tree species that can establish immediately after fire (Johnson 1996; Gutsell and Johnson 2002), whereas in forests with long stand‐replacing disturbance intervals, as originally hypothesized by Connell (1978), late‐successional species establish after local site environments have been modified by early‐successional species (Zhu et al. 2009; Zhang et al. 2014). Furthermore, disturbance and species richness relationships are dependent on whether understory species are considered. For example, Gosper et al. (2013) found a “U”‐shaped diversity – time since fire relationship with species from all forest strata considered because the species of subdominant functional types are suppressed under intensive resource competition during self‐thinning stage of stand development.

We found a positive linear species evenness–TSF relationship. This result is consistent with our hypothesis. The increase in species evenness with stand age is attributable to that the dominance of early‐successional species, that is, *Pinus banksiana*,* Populus* spp., decreases with stand development (Taylor and Chen 2011; Chen and Taylor 2012; Bergeron et al. 2014).

Previous studies have demonstrated that tree species diversity is strongly influenced by disturbance frequency alone (Brown and Gurevitch 2004; Gosper et al. 2013), which could be explained by controlling for a single factor, rather than multiple factors. However, in a large landscape other drivers (e.g., local site conditions) contribute to plant species diversity, and thus, we partitioned the effect of stand age from SDC, MAT, and MAP, and found that TSF and SDC are equally important factors in regulating tree species diversity, which is coherent with previous work (Zhang et al. 2014). SDC contributes significantly to tree species diversity (Huston 1993; Roberts and Gilliam 1995; Zhang et al. 2014), such that topographic soil moisture (Moeslund et al. 2013) and soil nutrient supply (Huston 1993; Cornwell and Grubb 2003) may directly control plant species diversity patterns. Previous studies have shown that climate exerts a potent influence on DDRs, particularly at regional and global levels (Francis and Currie 2003; Mayor et al. 2012); however, we observed that through TSF, SDC indirectly influenced tree species richness and evenness. These findings reflect the important role of SDC in influencing plant coexistence and therefore deserve attention in DDR (Roberts and Gilliam 1995; Zhang et al. 2014).

Theoretical studies often acknowledge that plant species coexistence is not attributable to a single mechanism, but rather, are the outcome of complex interacting mechanisms (Shea et al. 2004; Agrawal et al. 2007; Hughes et al. 2007), albeit empirical evidence is lacking. Thus, we have built on theoretical work by providing empirical insights on DDR, in which we explored multiple mechanisms underlying tree species diversity patterns using SEM. The SEM results demonstrated that in natural forest ecosystem subject to complex causal factors, several processes act simultaneously to influence plant species diversity. For example, we found a strong direct effect of SDC and MAT on TSF, while through TSF, MAT indirectly influenced species richness and evenness. These results provide a deeper understanding of DDR.

In conclusion, our results demonstrated that tree species richness decreases, while species evenness increases with time since fire in a boreal forest landscape. These results are attributed to the establishment of most trees species soon after fire and the decline of dominance of early‐successional, shade‐intolerant species with stand development. Moreover, our results demonstrated complex causal links between climate, local site condition, time since fire, and measures of species diversity in the boreal forest.

## Conflict of Interest

None declared.
